# Olfactory Receptors Modulate Physiological Processes in Human Airway Smooth Muscle Cells

**DOI:** 10.3389/fphys.2016.00339

**Published:** 2016-08-04

**Authors:** Benjamin Kalbe, Jürgen Knobloch, Viola M. Schulz, Christine Wecker, Marian Schlimm, Paul Scholz, Fabian Jansen, Erich Stoelben, Stathis Philippou, Erich Hecker, Hermann Lübbert, Andrea Koch, Hanns Hatt, Sabrina Osterloh

**Affiliations:** ^1^Department of Cell Physiology, Ruhr-University BochumBochum, Germany; ^2^Department of Internal Medicine III for Pneumology, Allergology, Sleep- and Respiratory Medicine, University Hospital BergmannsheilBochum, Germany; ^3^Department of Thoracic Surgery, Lungenklinik Merheim, Kliniken der Stadt KölnCologne, Germany; ^4^Department of Pathology and Cytology, Augusta-Kranken-AnstaltBochum, Germany; ^5^Thoraxzentrum Ruhrgebiet, Department of Thoracic Surgery, Evangelisches Krankenhaus HerneHerne, Germany; ^6^Department of Animal Physiology, Ruhr-University BochumBochum, Germany

**Keywords:** olfactory receptor, cytokines, smooth muscle cells, contraction, signaling

## Abstract

Pathophysiological mechanisms in human airway smooth muscle cells (HASMCs) significantly contribute to the progression of chronic inflammatory airway diseases with limited therapeutic options, such as severe asthma and COPD. These abnormalities include the contractility and hyperproduction of inflammatory proteins. To develop therapeutic strategies, key pathological mechanisms, and putative clinical targets need to be identified. In the present study, we demonstrated that the human olfactory receptors (ORs) OR1D2 and OR2AG1 are expressed at the RNA and protein levels in HASMCs. Using fluorometric calcium imaging, specific agonists for OR2AG1 and OR1D2 were identified to trigger transient Ca^2+^ increases in HASMCs via a cAMP-dependent signal transduction cascade. Furthermore, the activation of OR2AG1 via amyl butyrate inhibited the histamine-induced contraction of HASMCs, whereas the stimulation of OR1D2 with bourgeonal led to an increase in cell contractility. In addition, OR1D2 activation induced the secretion of IL-8 and GM-CSF. Both effects were inhibited by the specific OR1D2 antagonist undecanal. We herein provide the first evidence to show that ORs are functionally expressed in HASMCs and regulate pathophysiological processes. Therefore, ORs might be new therapeutic targets for these diseases, and blocking ORs could be an auspicious strategy for the treatment of early-stage chronic inflammatory lung diseases.

## Introduction

Olfactory receptors (ORs) represent the largest supergene family within the class of G protein-coupled receptors (GPCRs). They detect volatile odorant molecules by specific binding. Originally, olfactory sensory neurons (OSNs) within the olfactory epithelium (OE) were thought to exclusively express ORs (Buck and Axel, 1991). However, emerging data demonstrate that OR expression is not restricted to the OE but can be found in various human tissues (Feldmesser et al., 2006; Flegel et al., 2013). In the last decade, ORs were shown to regulate essential physiological functions, such as the regeneration of keratinocytes (Busse et al., 2014), sperm motility (Spehr et al., 2003), and the inhibition of prostate cancer (Neuhaus et al., 2009) and hepatocarcinoma (Maßberg et al., 2015) cell proliferation.

Canonical olfactory signaling is initialized by a conformational change of the OR after odorant binding. A heterotrimeric G protein (G_olf_) is activated (Jones and Reed, 1989; Belluscio et al., 1998), stimulating an adenylyl cyclase III (ACIII), which in turn generates cyclic adenosine monophosphate (cAMP; Bakalyar and Reed, 1990). This process leads to an opening of cyclic nucleotide-gated (CNG) channels, which conduct Ca^2+^ ions to the intracellular space, resulting in a depolarization of the OSN (Dhallan et al., 1990; Bradley et al., 1994). In non-chemosensory tissues, parts of this canonical pathway have been identified, but alternative signal transduction components have also been described (Spehr et al., 2011; Busse et al., 2014; Maßberg et al., 2015).

Abnormalities in human airway smooth muscle cell (HASMC) function and structure play critical roles in chronic inflammatory airway diseases, such as asthma, chronic obstructive pulmonary disease (COPD), and cystic fibrosis. One key feature in the pathophysiology of these diseases is the increase in airway lumen narrowing, which is caused by an increase in the mass of airway smooth muscles due to the hyperplasia and/or hypertrophy of HASMCs (Kim et al., 2008; Berair et al., 2013; Hirota and Martin, 2013; Sohal et al., 2013). Moreover, HASMCs secrete chemokines, cytokines, and growth factors in an inflammatory environment, thereby promoting the activation and migration of inflammatory cells, which consequently lead to airway and vascular remodeling (Knobloch et al., 2013). Additionally, the HASMCs of asthmatics often show hypercontractility. The contractility of these cells strongly depends on increases in the intracellular Ca^2+^ levels. To date, this Ca^2+^ increase has been attributed to the activation of a GPCR that initializes a phospholipase C-dependent pathway, leading to the release of Ca^2+^ ions from the intracellular sarcoplasmic reticulum stores via inositol triphosphate receptors (Sims et al., 1996; White et al., 2003). Taken together, the identification of mechanisms that affect the pathophysiological changes in HASMCs in patients with chronic inflammatory airway diseases are of eminent interest for the development of new clinical and therapeutic approaches. Because ORs regulate important physiological effects in several non-olfactory tissues and control the intracellular Ca^2+^ levels, we hypothesized that they might influence HASMC contractility and the secretion of inflammatory cytokines by HASMCs.

We demonstrated that different ORs are expressed at the mRNA and protein levels in HASMCs. In response to binding to specific ligands, they trigger intracellular Ca^2+^ increase via a cAMP-dependent pathway. Furthermore, OR activation specifically modulated HASMC contractility and the secretion of cytokines that are involved in airway inflammation in asthma, COPD, and other chronic lung diseases.

## Materials and methods

### HASMC isolation

HASMCs were dissected from lobar or main bronchus tissue obtained from patients undergoing lung resection for carcinoma of the bronchus, as described previously (Knobloch et al., 2009, 2013, 2014). Healthy tumor-free tissue was used for HASMC isolation. Briefly, the bronchial rings from the edges of the resected tissues that were tumor-free were placed in sterile Hank's buffered salt solution (HBSS; Sigma-Aldrich, St Louis, MO, USA, cat#-T-4674). Under sterile conditions, the smooth muscle layer was freed of adherent connective tissue: the smooth muscle was separated from the cartilage, and the epithelial layer and was cut into 1–3 mm^2^ pieces with a sterile scalpel blade. These tissue pieces were incubated in HBSS containing 10 mg/ml bovine serum albumin (BSA) and the enzymes collagenase (type XI, 1 mg/ml; Sigma-Aldrich cat#-C9407) and elastase (type I, 3.3 U/ml; Sigma-Aldrich cat#-E7885) for 30 min at 37°C in 5% CO_2_. After further removing the remaining connective tissue, the pieces were incubated in the aforementioned enzyme solution for 90–150 min, but the elastase content was increased to 15 U/ml. To separate the dispersed cells from the enzyme solution, the solutions were centrifuged (100 g, 5 min) at 4°C and then resuspended in Dulbecco's modified Eagle's medium (DMEM; Invitrogen, Karlsruhe, Germany, cat#-31885-023) containing 10% FCS (Sigma-Aldrich; cat#-N-4637), sodium pyruvate (1 mM; Invitrogen cat#-11360-039), L-glutamine (2 mM; Sigma-Aldrich cat#-G-7513), non-essential amino acids (1%; Invitrogen cat#-11140-035), penicillin (100 U/ml), streptomycin (100 μg/ml; Sigma-Aldrich cat#-P-4333), and amphotericin B (1.5 μg/ml; Sigma-Aldrich cat#-A-2942). This study was approved by the ethics committee of the University of Bochum (4257-12), Germany, and all donors gave their written consent.

### HASMC characterization and cultivation

HASMCs were characterized and cultivated as described previously (Knobloch et al., 2009, 2013, 2014). Briefly, the HASMC cellular suspension was placed in a tissue culture flask (25 cm^2^) with 8 ml of DMEM (supplemented as described above) and incubated at 37°C in 5% CO_2_. The culture medium was replaced after the initial 4–5 days and every 2–3 days thereafter. After 2–4 weeks, the cells reached confluence. Confluent cells were split 1:5 with trypsin (1% in HBSS) and further cultivated in 75 cm^2^ tissue culture flasks with 15 ml DMEM plus supplements. The HASMCs were characterized by positive immunostaining for calponin, smooth muscle α-actin, and myosin heavy chain (Knobloch et al., 2009, 2013, 2014). The cells were counted with a Neubauer haemocytometer; the cell viability was assessed by Trypan blue exclusion and was found to exceed 95%.

### HASMC count

Five thousand cells were seeded in wells of a 48-well-plate and serum-deprived for 16 h as described previously (Knobloch et al., 2013). The cells were then incubated with odorants or recombinant epidermal growth factor (EGF, R&D Systems, cat.: 236-EG; positive control) in low-glucose (1 g/l) DMEM supplemented with 1% FCS, 1 mM sodium pyruvate, 2 mM L-glutamine, 1% non-essential amino acids, 100 U/ml penicillin, 100 μg/ml streptomycin, and 1.5 μg/ml amphotericin B for 6 days. After incubation, the cells were stained with Trypan blue and counted in a Neubauer counting chamber as described before (Knobloch et al., 2008). The total number of viable cells and the ratio of dead to viable cells were determined.

### Total RNA isolation and reverse transcriptase (RT)-PCR

Total RNA was extracted from HASMCs using the RNeasy® Mini Kit (Qiagen, Hilden, Germany) according to the manufacturer's instructions. RNA concentration and quality (A260/A280 ratio) were analyzed using Spectrophotometer NanoDrop ND-1000 (Thermo Scientific, Waltham, MA, USA). After DNase I treatment with the TURBO DNA-free™ Kit (Thermo Scientific, Waltham, MA, USA), complementary DNA (cDNA) was synthesized using the iScript™ cDNA Synthesis Kit (Bio-Rad, Berkeley, CA, USA). For RT-PCR experiments, we used RNA controls (−RT) to exclude contamination with genomic DNA. RT-PCR was performed using GoTaq® qPCR Master Mix (Promega, Madison, WI, USA) in a volume of 20 μl with 10 pmols of each primer. The following temperature cycle profile was used: 5 min at 95°C followed by 40 cycles of 45 s at 95°C, 45 s at 60°C (OR1D2, OR2AG1 and ACTA2), 45 s at 72°C and a final extension of 10 min at 72°C. Following primer were used: OR2AG1 (forward: 5′-CCTTGTCACCTGCTCTTCCC-3′, reverse: 5′-AGCTAGCCATGATCCTTCCCT-3′), OR1D2 (forward: 5′-CCTGGCATCCCTGATTGCTA-3′, reverse: 5′-ATGGCATACGAAGCACAGTGAA-3′), ACTA2 (forward: 5′-CGGGACTAAGACGGGAATCCT-3′, reverse: 5′-CCATGTCGTCCCAGTTGGTG-3′).

### Immunocytochemical staining

HASMCs were cultured on 30 mm coverslips until 80% confluence was reached. After a washing step in PBS, the cells were fixed in ice-cold acetone for 5 min. To avoid non-specific antibody binding, the cells were blocked in 1% cold water fish gelatine (Sigma-Aldrich, USA) diluted in TBS with 0.05% Triton X-100 (Sigma-Aldrich, USA) before the incubation with primary antibody. Antibodies directed against the ORs OR1D2 (Novus Biologicals, Littleton, CO, USA) and OR2AG1 (Novus Biologicals, Littleton, CO, USA) as well as against the olfactory signaling components ACIII (Santa Cruz Biotechnology, Dallas, TX, USA), Gα_olf_ (Santa Cruz Biotechnology, Dallas, TX, USA), CNGA2 (Santa Cruz Biotechnology, Dallas, TX, USA), and CNGA4 (Santa Cruz Biotechnology, Dallas, TX, USA) were used. The cells were co-incubated with an antibody directed against smooth muscle actin (ACTA2; Abcam, Cambridge, UK) to verify that all cells are HASMCs. To analyse the specificity of OR1D2 and OR2AG1 antibodies, these receptors were overexpressed in HANA3A cells. For that, pCI plasmids containing the sequence of the OR-coding region were transfected (OR1D2 NCBI Accession No.: NM_002548.2; OR2AG1 NCBI Accession No.: NM_001004489.2). As a control, cells were mock transfected with pCI plasmid. Fluorophore-coupled secondary antibodies (Alexa Fluor 488 or 546 nm; Thermo Fisher Scientific, USA) were used, and the cells were coated with Prolong Antifade Gold (Life Technologies, USA). The fluorescent signals were detected using a confocal microscope (Zeiss LSM 510 Meta, Germany) with a 40x oil immersion objective and the Leica Application Suite software (LAS, Leica, Germany). The images where processed using Corel Draw X5 (Corel, USA).

### Immunohistochemical staining of lung tissue

Paraffin-embedded human lung tissue was deparaffinized using Roti®-Histol (Roth, Karlsruhe, Germany) and a subsequent dehydration step with isopropanol was conducted. The tissue was rehydrated with an ethanol-series and washed twice with 0.01 M PBS buffer. Next, antigen retrieval and permeabilization steps were performed. To prevent unspecific primary antibody binding, sections were blocked in 5% normal serum for 10 min. Primary antibody (OR1D2 or OR2AG1, dilution: 1:50) was incubated in 0.01 M PBS buffer at 4°C over night. Sections were washed again with 0.01 M PBS buffer and horse radish peroxidase (HRP)-coupled secondary antibody (anti-rabbit) was incubated for 45 min (dilution: 1:1000). VECTASTAIN®Elite avidin/biotin (ABC)-based Kit (Vector laboratories, Burlingame, USA) was used after the manufacturer's recommendations. 3,3′-diaminobenzidine was incubated until a sufficient staining developed and the reaction was stopped with 0.1 M PB buffer on 4°C. Immunohistochemical staining was detected with an Olympus BX 43 microscope (10x objective). Lung tissue was obtained from patients undergoing a surgical intervention during a carcinoma resection. This was conducted according to the Declaration of Helsinki and all patients gave their written consent.

### Western blotting

Whole-protein lysate was extracted from sedimented HASMCs, followed by solubilization in an appropriate volume of RIPA buffer, mechanical homogenization and a final centrifugation (1000 g for 10 min). A sample of the whole protein fraction was collected and prepared in Laemmli's buffer for western blot analysis. For the membrane preparation, whole protein lysate was subjected to ultracentrifugation (35,000 g for 2 h). The precipitate and a sample of the supernatant were dissolved in Laemmli's buffer for further western blot analyses. Samples of all fractions were loaded on SDS gels, blotted on nitrocellulose membranes (GE healthcare, UK) and incubated with the aforementioned antibodies after a blocking step in 50% casein (50% TBS buffer, 50% casein in TBS, Thermo Scientific, USA). Primary antibodies against OR1D2, OR2AG1, ACIII, Gα_olf_, CNGA2, and CNGA4 were diluted 1:250 in 25% casein (75% TBS buffer, 25% casein). Horseradish peroxidase (HRP)-coupled secondary antibodies were used (goat anti-rabbit, rabbit anti-goat, Bio-Rad, UK) for immunodetection. ECL western blotting detection reagent (GE Healthcare, UK) was used for the detection step, and the chemiluminescence was imaged using a Fusion-SL 3500-WL (Vilber Lourmat, Germany).

### Calcium imaging

HASMCs cultured in 35 mm cell culture dishes were incubated with 3 μM fura-2-acetoxymethyl ester (Molecular Probes) for 30 min at 37°C. The growth medium was exchanged with extracellular solution, and fluorometric imaging was performed as previously described (Spehr et al., 2003; Busse et al., 2014; Maßberg et al., 2015). Depending on the experimental approach, the cells were exposed to 0.1–3 mM amyl butyrate (Henkel, Germany), 0.0001–100 μM bourgeonal (Givaudan, Switzerland), or 300 μM of the other OR1D2 agonists [Lilial (Givaudan, Switzerland) and 4-PBA (Givaudan, Switzerland)] using a specialized microcapillary application system. All substances were pre-diluted in DMSO (maximal final concentration 0.1%) and dissolved in extracellular solution to the desired concentration. Inhibitors and antagonists were used as published in previous studies and either co-applied [L-cis-diltiazem 100 μM (Abcam, UK; Leung et al., 2010; Busse et al., 2014) and undecanal 200 μM (Sigma-Aldrich, USA)] or pre-incubated [MDL12330A 50 μM (Enzo Life Sciences, USA; Grosmaitre et al., 2006) and SQ22536 200 μM (Enzo Life Sciences, USA; Benbernou et al., 2011)]. Cells were washed before and after application of the agonists or inhibitors. Data from the calcium imaging experiments were processed using the Leica Application Software (LAS, Leica, Germany). The amplitudes and EC50-values were calculated using Sigma Plot (Systat, USA). Concentration-response curves were fitted with the 4-parameter Hill model using Sigma Plot.

### cAMP-Glo assay

HASMCs were seeded on poly-d-lysine-coated 96-well-plates (NUNC) at a density of 1.5 × 10^4^ cells/well. Elevated cAMP level was measured using the cAMP-Glo™ Assay (Promega) according to the manufacturer's recommendations and described elsewhere (Maßberg et al., 2015). The data were normalized to the DMSO (0.1%) control.

### Contraction assay

HASMC contraction experiments were performed with a collagen gel-based assay kit (Cell Contraction Assay; Cell Biolabs CBA-201) according to the manufacturer's protocol.

### Cytokine measurements

For stimulation, HASMCs were plated at equal density in 6-well-plates. Before stimulation, sub-confluent cell monolayers (~80% confluence) in six-well-cell culture plates were deprived of serum for 24 h in serum-free and low-glucose (1 g/l) DMEM (Invitrogen; cat#-41966-029) supplemented containing 1 mM sodium pyruvate, 2 mM L-glutamine, 1% non-essential amino acids, 100 U/ml penicillin, 100 μg/ml streptomycin, 1.5 μg/ml amphotericin B, 1 mM insulin (Sigma-Aldrich cat#-I1882), 5 mg/ml, apo-transferrin (Sigma-Aldrich cat#-T1147) and 100 μM ascorbic acid (Sigma-Aldrich cat#-A4403), as described previously (Knobloch et al., 2009, 2013, 2014).

HASMCs were stimulated in serum-free and low-glucose (1 g/l) DMEM supplemented with 1 mM sodium pyruvate, 2 mM L-glutamine, 1% non-essential amino acids, 100 U/ml penicillin, 100 μg/ml streptomycin, and 1.5 μg/ml amphotericin B. To measure the levels of cytokines in the supernatant, untreated HASMCs at passages 2–7 were stimulated with either bourgeonal (100 μM), amyl butyrate (100 μM), or undecanal (200 μM) for 24, 48, or 72 h. Cell viability was determined by Trypan blue staining. None of the drugs induced cell death in HASMCs at the conditions used in this study. The IL-8 and GM-CSF concentrations in the cell culture supernatants were measured with an ELISA (Duo Sets DY208, DY215; R&D Systems) according to standard protocols (Knobloch et al., 2009, 2013, 2014).

### Statistical analysis

All results were tested for normality and equal variance. Data that passed the equal variance and normality tests were subjected to a two-tailed unpaired *t*-test. Data that failed the aforementioned tests were subjected to a Mann-Whitney U-test. All values represent the mean ± standard error of the mean (SEM) of at least three independent experiments. In all figures, the significance of differences is represented as follows: ^*^p < 0.05, ^**^p < 0.01, and ^***^p < 0.001.

## Results

### Specific odorants elicit an intracellular Ca^2+^ increase via olfactory receptors in HASMCs

**Figure 1:**
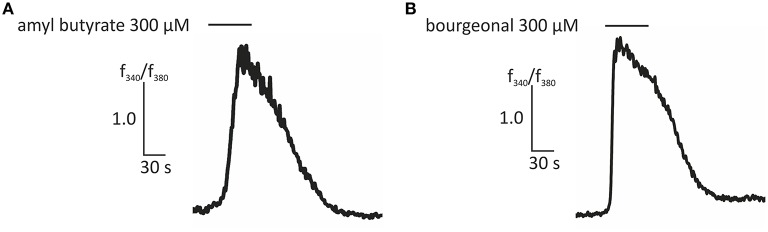
**Representative traces of ratiometric Ca^**2+**^ imaging experiments showing an increase in intracellular Ca^**2+**^ evoked by amyl butyrate (300 μM; A) and bourgeonal (300 μM; B)**. Bars indicate the application duration.

**Figure 2:**
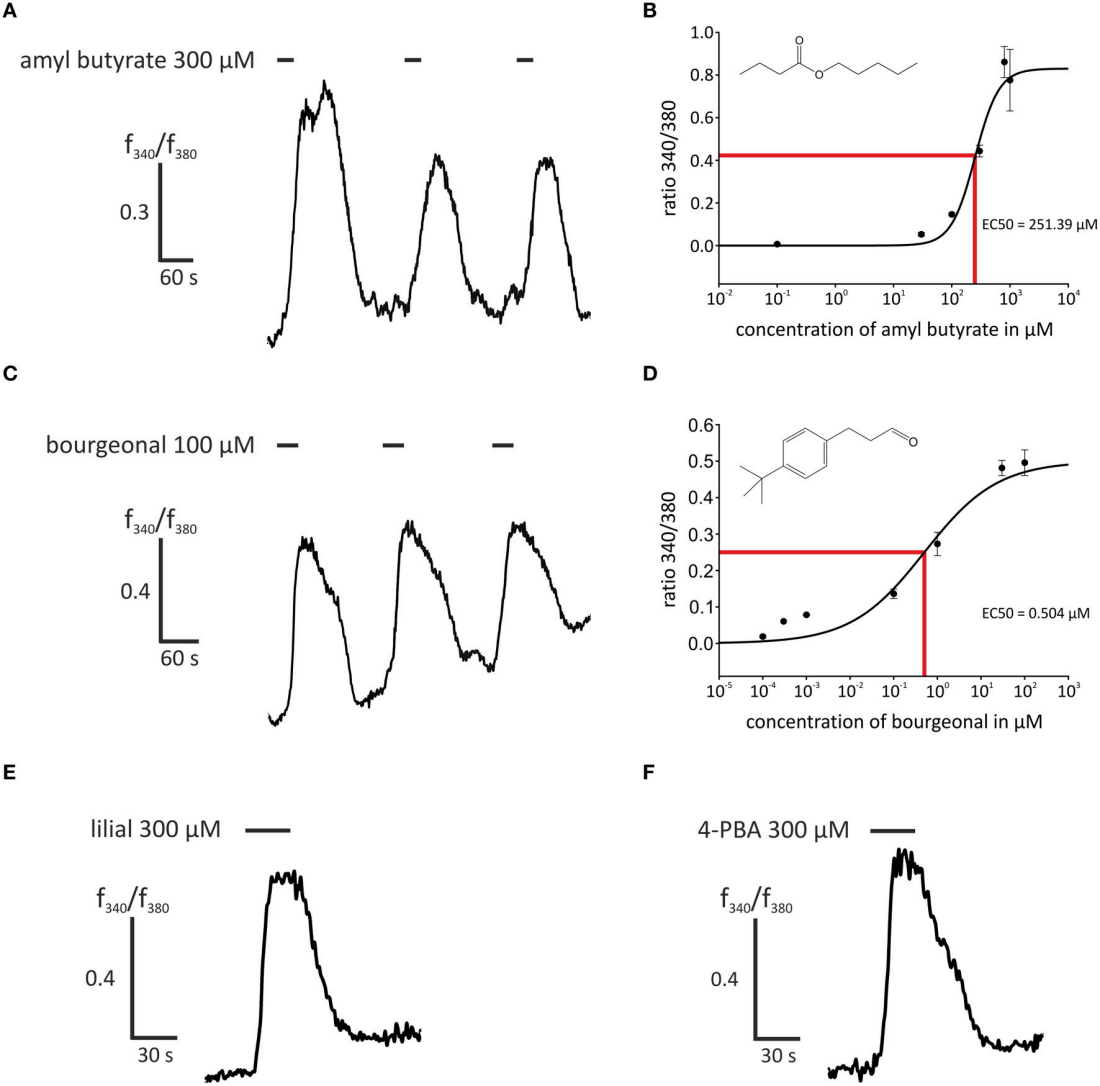
**Stimulation with agonists of OR2AG1 and OR1D2 led to an intracellular Ca^**2+**^ increase in HASMCs. (A)** Repetitive stimulation with amyl butyrate (300 μM, duration: 30 s) elicited a reproducible transient increase in intracellular Ca^2+^ measured with the ratiometric Ca^2+^ indicator FURA-2AM. **(B)** Amyl butyrate, an agonist for OR2AG1, activated HASMCs in a dose-dependent manner with an EC50 of 253.39 μM (*N* = 6). **(C)** The application of the OR1D2 agonist bourgeonal (100 μM, duration: 30 s) led to an increase in intracellular Ca^2+^, and repetitive stimulation exerted a reproducible effect. **(D)** Bourgeonal was able to activate HASMCs in a dose-dependent manner with an EC50 of 0.5043 μM (*N* = 3–5). **(E,F)** The application of the OR1D2 agonists lilial (300 μM, duration: 30 s; **E)** and 4-phenylbutyrate (4-PBA; 300 μM, duration: 30 s; **F**) led to an intracellular Ca^2+^ increase in ratiometric Ca^2+^ imaging experiments. Bars indicate the application duration. Error bars represent the ± SEM of three to four independent experiments.

In this study, we first aimed to characterize the odor-dependent activation of HASMCs. Because OR activation leads to a Ca^2+^ influx in OR neurons and Ca^2+^ initiates the contraction of HASMCs, we investigated the intracellular Ca^2+^ levels after receptor activation using fluorometric calcium imaging. First, we stimulated HASMCs with either amyl butyrate [specific ligand for OR2AG1 (Mashukova et al., 2006)] or bourgeonal [specific ligand for OR1D2 (Spehr et al., 2003)], which both induced a strong transient intracellular Ca^2+^ increase (Figure 1). In addition, amyl butyrate (300 μM) was repetitively applied for 30 s and elicited reproducible strong Ca^2+^ signals in most cells (Figure 2A). We analyzed the concentration-dependence of the cytosolic Ca^2+^ levels after stimulation with amyl butyrate in the HASMCs and calculated an EC50-value of 251.39 μM (Figure 2B).

Moreover, we examined the activation of OR1D2 in more detail by applying the known ligands bourgeonal (100 μM), lilial (300 μM), and 4-PBA (300 μM), which all induced reproducible strong Ca^2+^ responses in most HASMCs (Figures 2C,E,F). The repeated application of bourgeonal led to recurrent Ca^2+^ signals (Figure 2C). We monitored the concentration-dependence of the Ca^2+^ responses to the application of bourgeonal and measured an EC50 of 0.5 μM (Figure 2D). To exclude any bias of the dose-response curves due to shifted baselines or desensitization after repetitive stimulation, different odorant concentrations were administered in single applications.

### Olfactory receptors and signaling proteins are expressed at the RNA and protein levels in HASMCs

**Figure 3:**
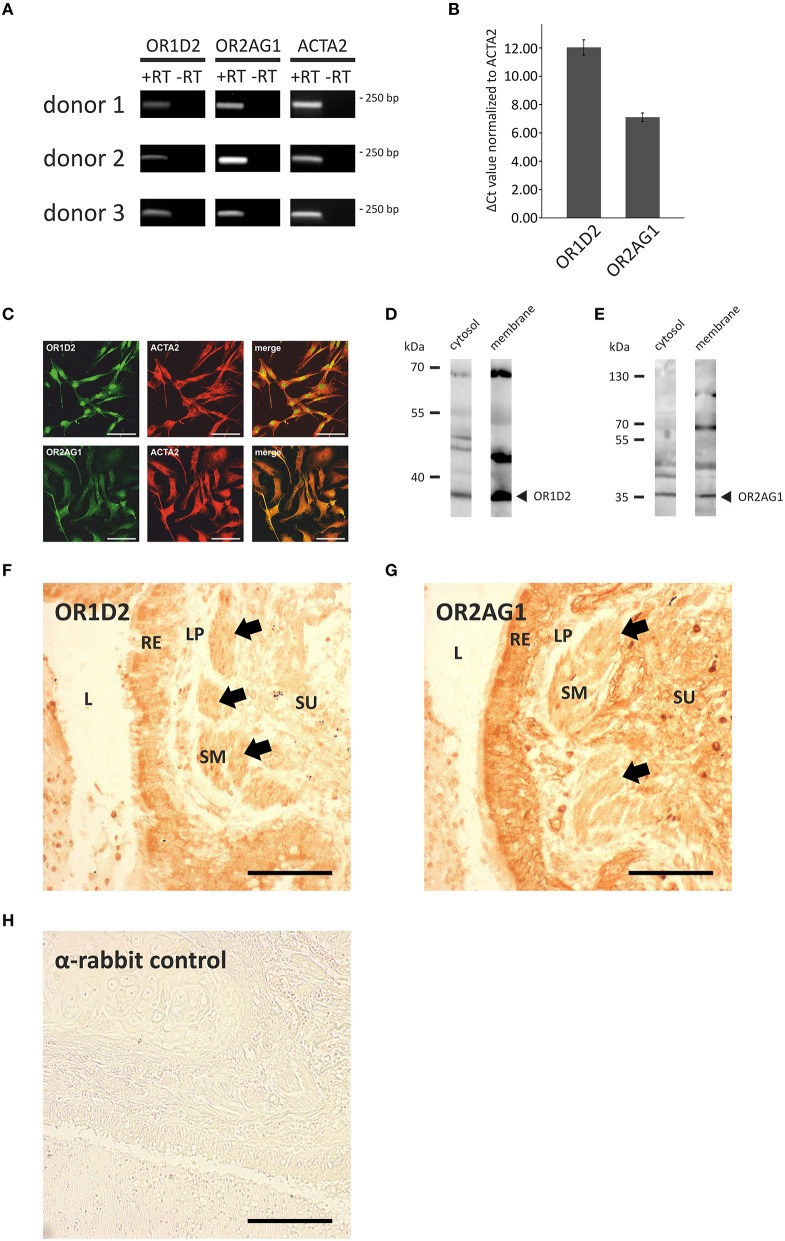
**Analysis of the expression of ORs and olfactory signaling components at the transcript and protein level. (A,B)** qPCR analysis with cDNA of three different HASMC donors using specific OR primers. PCR products for OR1D2, OR2AG1, and the smooth muscle marker ACTA2 were detected in an agarose gel at ~250 bp **(A)**. ΔCt-values were calculated and normalized to the ACTA2 Ct-value (*N* = 3). The transcript level of OR2AG1 was higher than that of OR1D2 **(B)**. Error bars represent the ±SEM of at least three independent experiments. **(C)** The immunocytochemical staining of HASMCs showed that OR1D2 and OR2AG1 are expressed at the protein level. The cells were co-stained with ACTA2 as a marker of HASMCs. Scale bars: 100 μm. **(D,E)** Western blot experiments using the cytosolic and membrane protein fractions of HASMCs showed specific bands for OR1D2 (~35 kDa; **D)** and OR2AG1 (~35 kDa; **E)** in all fractions. **(F–H)** Immunohistochemical 3,3′-diaminobenzidine (DAB) staining of human lung tissue with OR1D2 **(F)** and OR2AG1 **(G)** antibody. A detail of a bronchus can be seen in both images. L, lumen; RE, respiratory epithelium; LP, lamina propria; SM, smooth muscle layer; SU, submucosa. Specific staining was observed in the apical part of the RE and in the SM **(F)** as well as in the RE, SM, and cells of the SU **(G)**. **(H)** Secondary antibody (anti-rabbit) negative control. Arrows indicate staining of the SM. Scaling bar: 200 μm.

**Figure 4:**
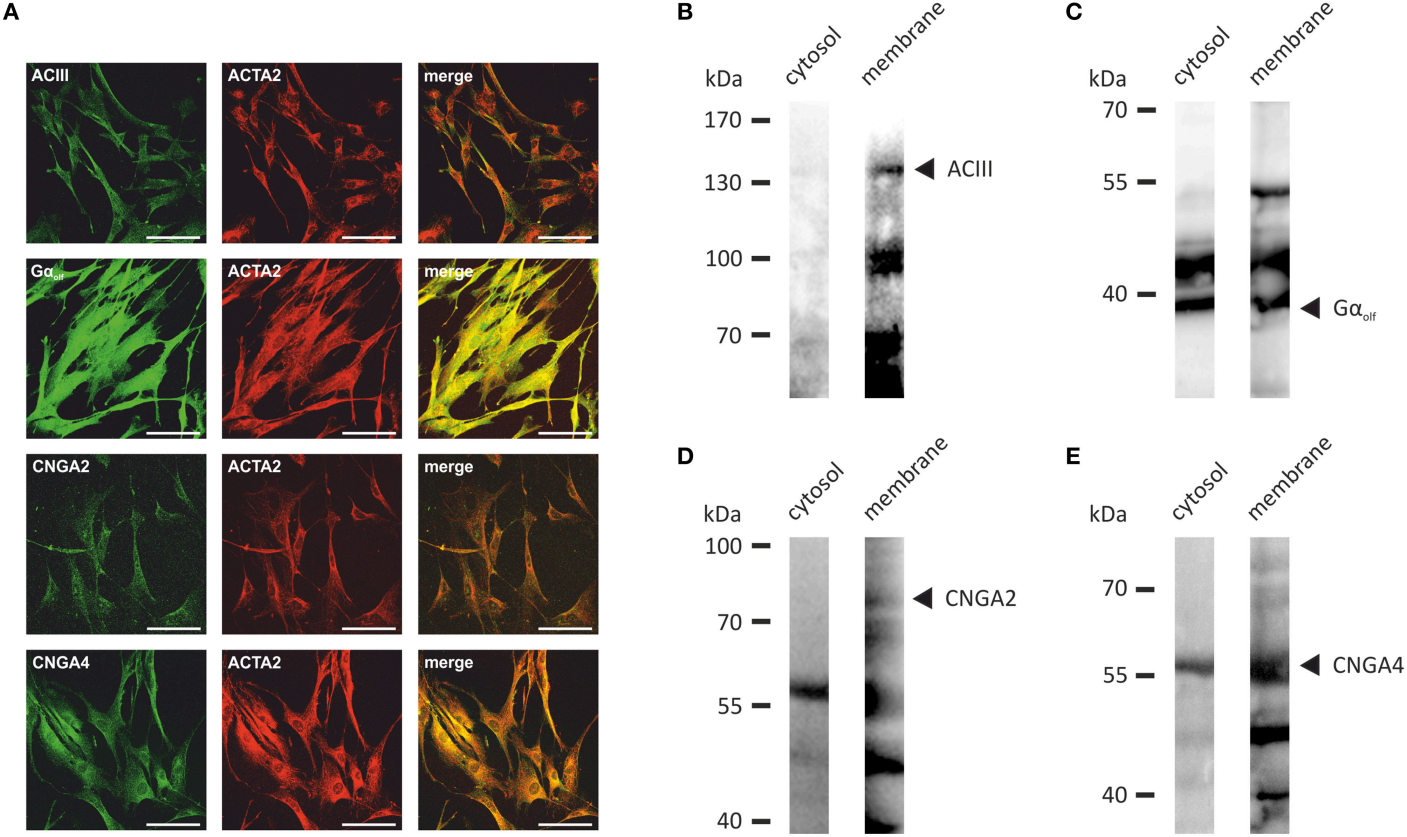
**(A)** The immunocytochemical staining of HASMCs showed that the olfactory signaling proteins ACIII, G_olf_, CNGA2, and CNGA4 are expressed at the protein level. The cells were co-stained with ACTA2 as a marker for HASMCs. Scale bars: 100 μm. **(B–E)** Specific bands for ACIII (~130 kDa; **B)**, Gα_olf_ (~40 kDa; **C)**, CNGA2 (~80 kDa; **D**), and CNGA4 (~60 kDa; **E)** were detected in the membrane fraction of HASMCs via western blot analysis of the membrane and cytosolic fractions.

Next, we investigated the transcript levels of these receptors in the HASMCs of three different donors via RT-qPCR and found specific amplicons at a size of ~250 bp for OR1D2, OR2AG1, and the smooth muscle-specific actin ACTA2 (Figure 3A). We calculated the ΔCt-value normalized to ACTA2 and observed a higher transcript level of OR2AG1 in relation to OR1D2 (Figure 3B). To characterize the protein expression of OR and signaling factors, immunocytochemical staining was performed using specific antibodies. We observed the expression of the human ORs OR1D2 and OR2AG1 at the protein level. ACTA2 was used as a HASMC marker (Figure 3C). We verified these results using western blot experiments with the cytosolic and membrane-enriched fractions of HASMC (Figures 3D,E). We detected specific proteins bands for the ORs OR1D2 (35 kDa; Figure 3D) and OR2AG1 (35 kDa; Figure 3E), as well as signals at the protein weight of receptor dimers. The protein abundance was stronger in the membrane fraction than in the cytosolic fraction. In addition, OR1D2 and OR2AG1 proteins were also detected in human biopsies using immunohistochemical staining (Figures 3F–H). There, OR1D2 and OR2AG1 were localized in the bronchial epithelium, in smooth muscle cells and cells of the submucosa. To analyse the specificity of OR1D2 and OR2AG1 antibodies in the immunocytochemical staining, we transfected HANA3A cells with OR1D2- and OR2AG1-carrying plasmids and observed a specific stain of HANA3A (Supplementary Figures 2A,B). Mock-transfected cells showed no stain with either OR1D2 or OR2AG1 antibody (Supplementary Figures 2C,D). Additionally, we specifically stained cells for the olfactory signal transduction proteins ACIII, Golf, CNGA2, and CNGA4 (Figure 4A). Negative controls using only secondary fluorophore-coupled antibody remained unstained (Supplementary Figure 1). Furthermore, we confirmed the expression of ACIII (Figure 4B), Gα_olf_ (Figure 4C), CNGA2 (Figure 4D), and CNGA4 (Figure 4E) within the membrane and cytosol of the cell via western blot analysis.

### Odor-specific intracellular Ca^2+^ increase in HASMCs is mediated by the canonical olfactory signaling pathway

**Figure 5:**
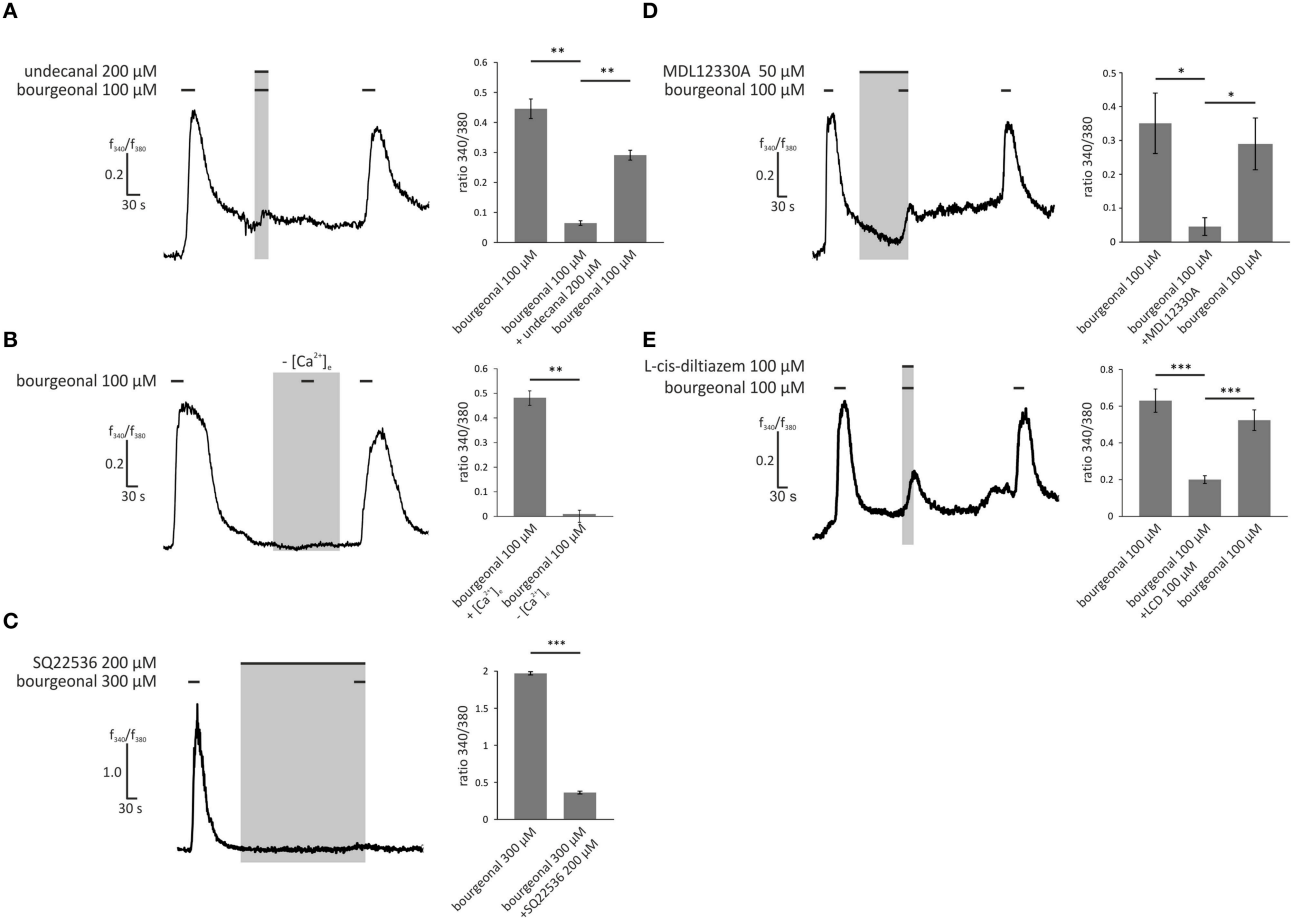
**Bourgeonal-induced Ca^**2+**^ increase in HASMCs is dependent on the extracellular Ca^**2+**^ concentration, production of cAMP, and opening of CNG channels**. **(A)** The bourgeonal-specific antagonist undecanal significantly inhibited the activation of OR1D2 and, thereby, Ca^2+^ increases in HASMCs. Bourgeonal (100 μM) was first applied for 30 s. After a 3 min wash in Ringer's solution, undecanal (200 μM) and bourgeonal (100 μM) were co-applied for 30 s. The cells were again washed with Ringer's solution, followed by an application of bourgeonal (100 μM) for 30 s. The undecanal-induced inhibition was reversible (*N* = 5). **(B)** Bourgeonal (100 μM) was applied for 30 s in Ringer's solution containing 2 mM extracellular Ca^2+^ or in a Ca^2+^-free condition with 5 mM EGTA. Under Ca^2+^ free conditions, the cytosolic Ca^2+^ increase was completely abolished (*N* = 6). **(C)** Bourgeonal (100 μM) was applied for 30 s after a 5 min incubation with the adenylyl cyclase inhibitor SQ22536 (200 μM). Ca^2+^ influx was significantly reduced after incubation with SQ22536 (*N* = 3). **(D)** MDL12330A (50 μM), an adenylyl cyclase inhibitor, was applied for 2 min, followed by co-application with bourgeonal (100 μM, duration: 30 s), which significantly inhibited bourgeonal-induced Ca^2+^ increase. This effect was reversible after a 4 min washing step (*N* = 4). **(E)** The CNG channel inhibitor L-cis-diltiazem (100 μM) was co-applied with bourgeonal (100 μM) for 30 s and significantly reduced the Ca^2+^ influx induced by bourgeonal. The inhibitory effect of L-cis-diltiazem was reversible after a wash-out (*N* = 6). The bars of all experiments indicate the stimulus duration. All error bars represent the ±SEM of at three to six independent experiments. Significance was tested with an unpaired two-sample Student's *t*-test or a Mann-Whitney *U* test. ^*^p < 0.05, ^**^p < 0.01, and ^***^p < 0.001.

To identify the signaling pathways that become activated by odors in HASMCs, we performed pharmacological blocking experiments and analyzed the intracellular Ca^2+^ levels. To confirm the involvement of OR1D2 in bourgeonal-induced Ca^2+^ responses, we co-applied the known antagonist undecanal (Spehr et al., 2003; 200 μM) together with bourgeonal (100 μM). Undecanal significantly reduced the bourgeonal-induced Ca^2+^ response, and this effect was reversible after wash-out (Figure 5A). After removing the extracellular free Ca^2+^ ions using the Ca^2+^ chelator EGTA (5 mM), the bourgeonal-induced Ca^2+^ increase was completely abolished (Figure 5B), indicating the importance of extracellular Ca^2+^. Next, the adenylyl cyclase inhibitors SQ22536 (200 μM) and MDL12330A (50 μM) were used to investigate the contribution of the cAMP-producing enzyme in this signaling cascade. In the presence of both blockers, the OR-dependent Ca^2+^ increase was significantly reduced (Figures 5C,D). We performed cAMP assays and validated the production of cAMP after stimulation with bourgeonal (Supplementary Figure 3B). This effect was significantly inhibited by undecanal (Supplementary Figure 3C). Moreover, the CNG channel inhibitor L-cis-diltiazem (100 μM) was used to analyse the involvement of CNG channels. The bourgeonal-elicited Ca^2+^ increase was significantly reduced in the presence of this blocker (Figure 5E).

**Figure 6:**
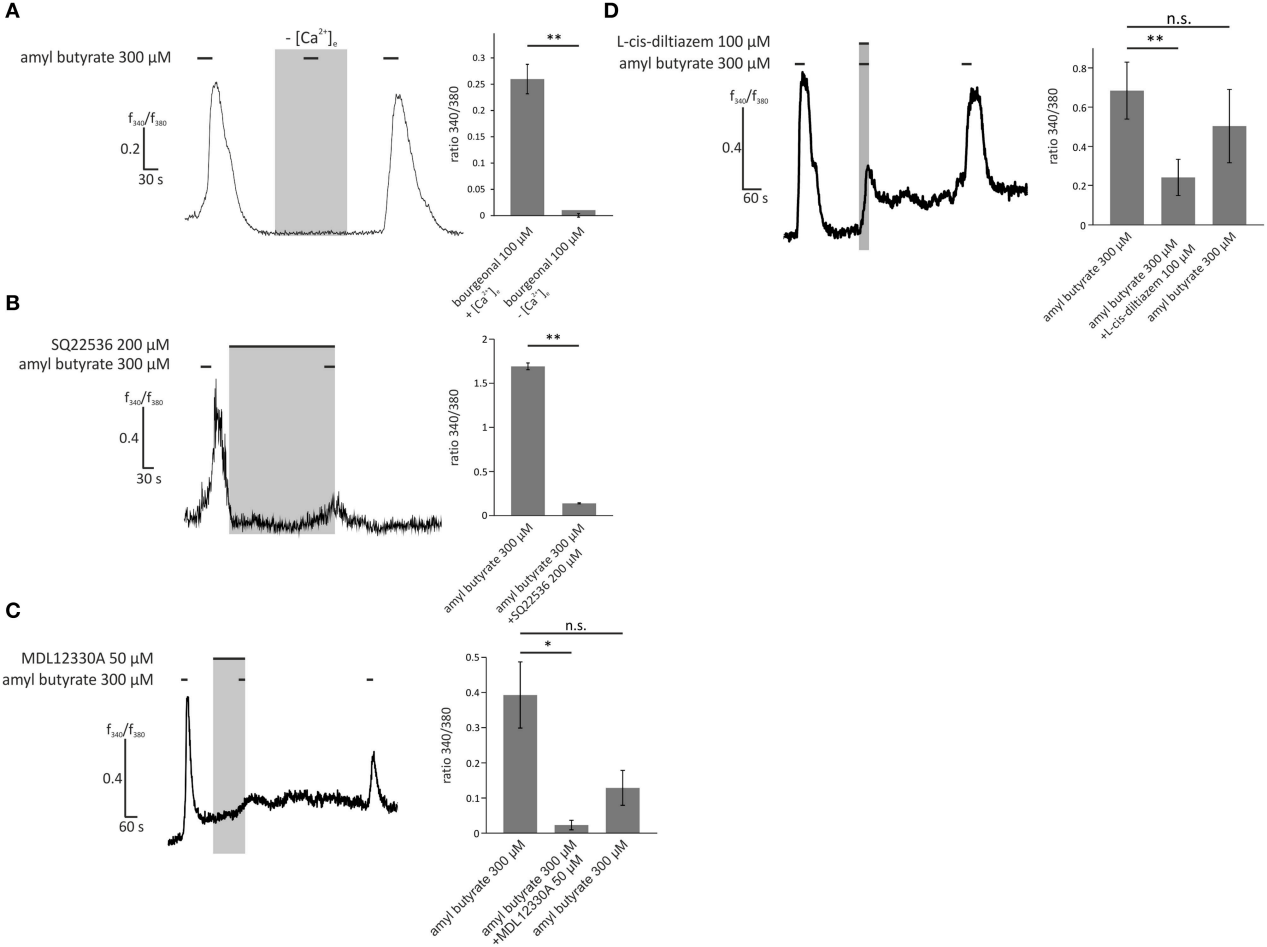
**Activation of HASMCs by the OR2AG1-specific agonist amyl butyrate led to an influx of Ca^**2+**^ dependent on cAMP production and opening of CNG channels. (A)** Amyl butyrate (300 μM) was applied for 30 s in Ringer's solution with 2 mM extracellular Ca^2+^ or in a Ca^2+^-free Ringer's solution with 5 mM EGTA. Under Ca^2+^ free conditions, the cytosolic Ca^2+^ increase was completely abolished (*N* = 3). **(B)** Amyl butyrate (300 μM) was applied for 30 s after a 5 min incubation with the adenylyl cyclase inhibitor SQ22536 (200 μM). Ca^2+^ influx was significantly reduced after incubation with SQ22536 (*N* = 3). **(C)** A 2 min incubation with MDL12330A (50 μM), an adenylyl cyclase inhibitor, and a following co-application with amyl butyrate (300 μM, duration: 30 s) significantly inhibited amyl the butyrate-induced Ca^2+^ increase (*N* = 4). **(D)** The CNG channel inhibitor L-cis-diltiazem (100 μM) was co-applied with amyl butyrate (300 μM) for 30 s and significantly reduced the Ca^2+^ influx induced by amyl butyrate. The inhibitory effect of L-cis-diltiazem was reversible after a wash-out (*N* = 5). Bars of all experiments indicate the stimulus duration. All error bars represent the ±SEM of at three to five independent experiments. Significance was tested with an unpaired two-sample Student's *t*-test or a Mann-Whitney *U* test. ^*^p < 0.05, ^**^p < 0.01.

To examine the dependence of the OR2AG1-specific pathway in HASMCs on the aforementioned olfactory signaling proteins, experiments with the specific OR2AG1 agonist amyl butyrate (300 μM) were performed. The withdrawal of extracellular free Ca^2+^ via EGTA abolished the amyl butyrate-induced Ca^2+^ responses (Figure 6A). The adenylyl cyclase blockers SQ22536 (Figure 6B) and MDL12330A (Figure 6C) both significantly reduced the amyl butyrate-induced Ca^2+^ signals. Again, we verified the production of cAMP upon stimulation with amyl butyrate by a cAMP assay (Supplementary Figure 3A). In the presence of L-cis-diltiazem, the amyl butyrate-triggered Ca^2+^ currents were also strongly diminished (Figure 6D). Both effects were completely reversible after washing out the respective inhibitor. Interestingly, we observed that the histamine-induced Ca^2+^ response of HASMCs can be inhibited by the co-application of either amyl butyrate or bourgeonal (Supplementary Figure 4).

### Olfactory receptor activation affects the contractility of HASMCs

**Figure 7:**
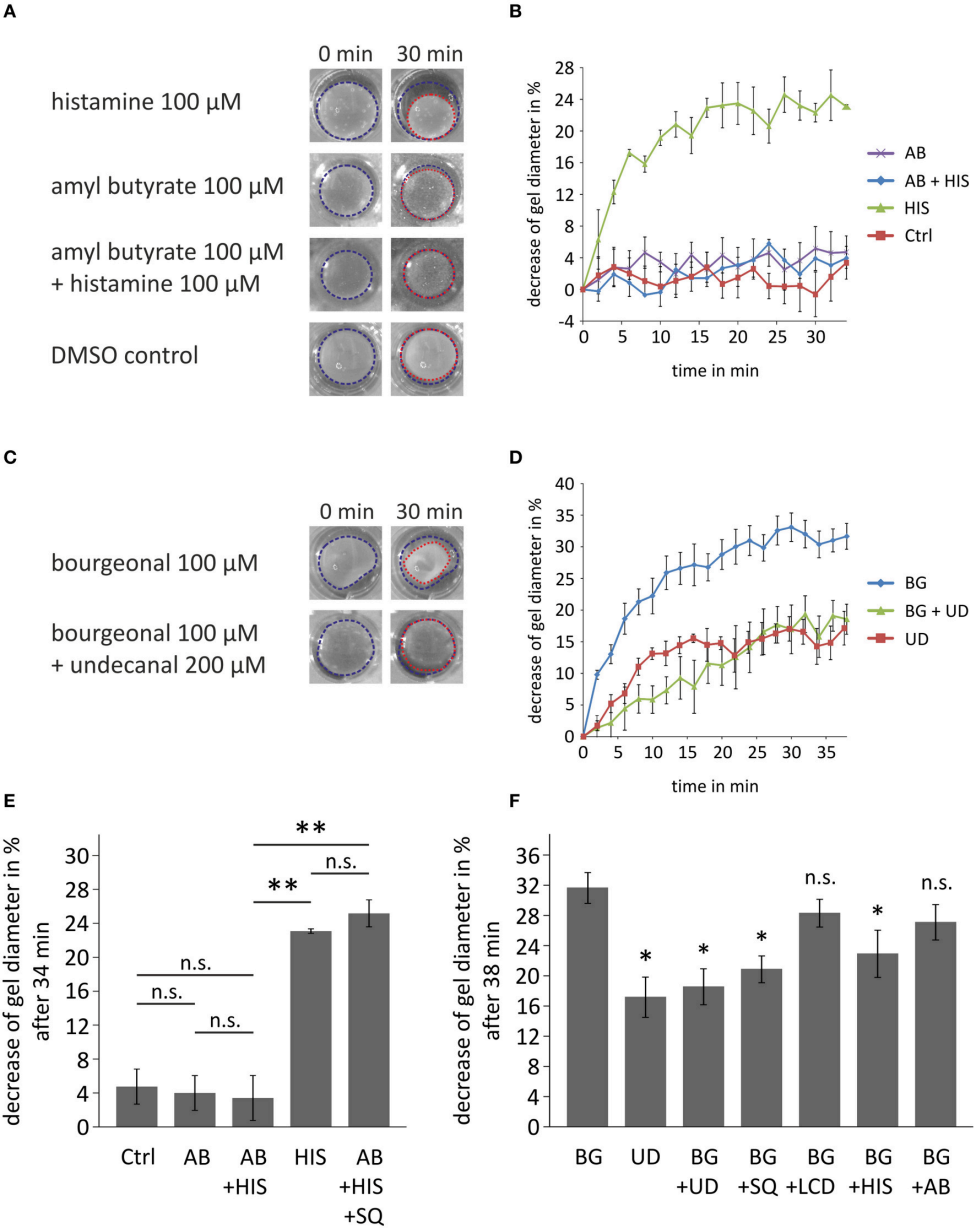
**Cell contraction assays demonstrating the physiological relevance of the activation of ORs in HASMCs**. **(A,B,E)** Contraction analysis after the incubation of collagen gel-embedded HASMCs with histamine (HIS; 100 μM), amyl butyrate (AB; 100 μM), and co-incubation with histamine (100 μM) and amyl butyrate (100 μM). The gel diameter decrease is exemplarily shown after 30 min of incubation with drug **(A)**. As a control, cells were incubated with DMEM + 0.1% DMSO (DMSO control, Ctrl). Histamine induced a significant decrease in the gel diameter when incubated with HASMC collagen gels for 34 min **(B,E)**. Amyl butyrate itself did not alter the gel diameter under these conditions and produced no significant changes in relation to the medium control. The co-incubation of amyl butyrate and histamine resulted in a non-significant decrease in relation to the medium control but a significant inhibition of the contraction induced by histamine alone (*N* = 3). The AC inhibitor SQ22536 (SQ; 200 μM) inhibited the amyl butyrate-induced reduction of histamine-mediated contraction (*N* = 3). **(C,D,F)** Analysis of the effects of bourgeonal on HASMC contraction. The gel diameter decrease is exemplarily shown after 30 min of incubation with either bourgeonal (BG; 100 μM), undecanal (UD; 200 μM) or bourgeonal (100 μM), and undecanal (BG + UD; 200 μM;) **(D)**. Bourgeonal (100 μM) induced a noticeable decrease in the gel diameter **(D,F)**. Undecanal itself induced a weaker contraction of HASMCs. The bourgeonal-induced effect was significantly inhibited by the co-incubation of the bourgeonal-specific antagonist undecanal (200 μM; *N* = 4; **F**). The effect of bourgeonal was also inhibited by SQ22536 (SQ; 200 μM) and histamine (100 μM), but not L-cis-diltiazem (LCD) or amyl butyrate (*N* = 3). Blue and red dotted lines indicate the gel area. All error bars represent the ±SEM of either three or four experiments. Significance was tested with an unpaired two-sample Student's *t*-test. In **(F)** significance is tested compared to bourgeonal-induced decrease of gel diameter. ^*^p < 0.05, ^**^p < 0.01.

Next, we investigated the physiological importance of OR-activation in HASMCs with a collagen gel-based contraction assay. The gel percentage contraction in response to histamine (100 μM) was measured in the presence of amyl butyrate (100 μM; activation of OR2AG1) after 34 min of incubation (Figure 7A). The relative contraction strength was estimated by calculating the decrease in the gel diameter (Figure 7B). A medium control was performed to exclude the effects of spontaneous or mechanically triggered contraction. The contraction of histamine-exposed (100 μM) HASMCs was significantly stronger than those of cells treated with 100 μM amyl butyrate and the DMSO control (Figure 7C). The observed effect during histamine incubation was completely abolished after co-treatment with amyl butyrate (100 μM), indicating that OR2AG1 activation inhibits the contraction of HASMCs. This effect could be inhibited by the co-stimulation with the AC inhibitor SQ22536 (Figure 7E).

In contrast, the activation of OR1D2 via bourgeonal (100 μM) evoked an increase in HASMC contraction that was comparable to the histamine-induced effect. Bourgeonal-triggered cell contractility was significantly inhibited by co-applying the specific antagonist undecanal (200 μM; Figures 7D,F). Bourgeonal-induced contraction was also inhibited by the AC inhibitor SQ22536 and co-application with histamine, but not by L-cis-diltiazem or amyl butyrate (Figure 7F).

### OR1D2 activation induces the release of the cytokines IL-8 and GM-CSF in HASMCs

**Figure 8:**
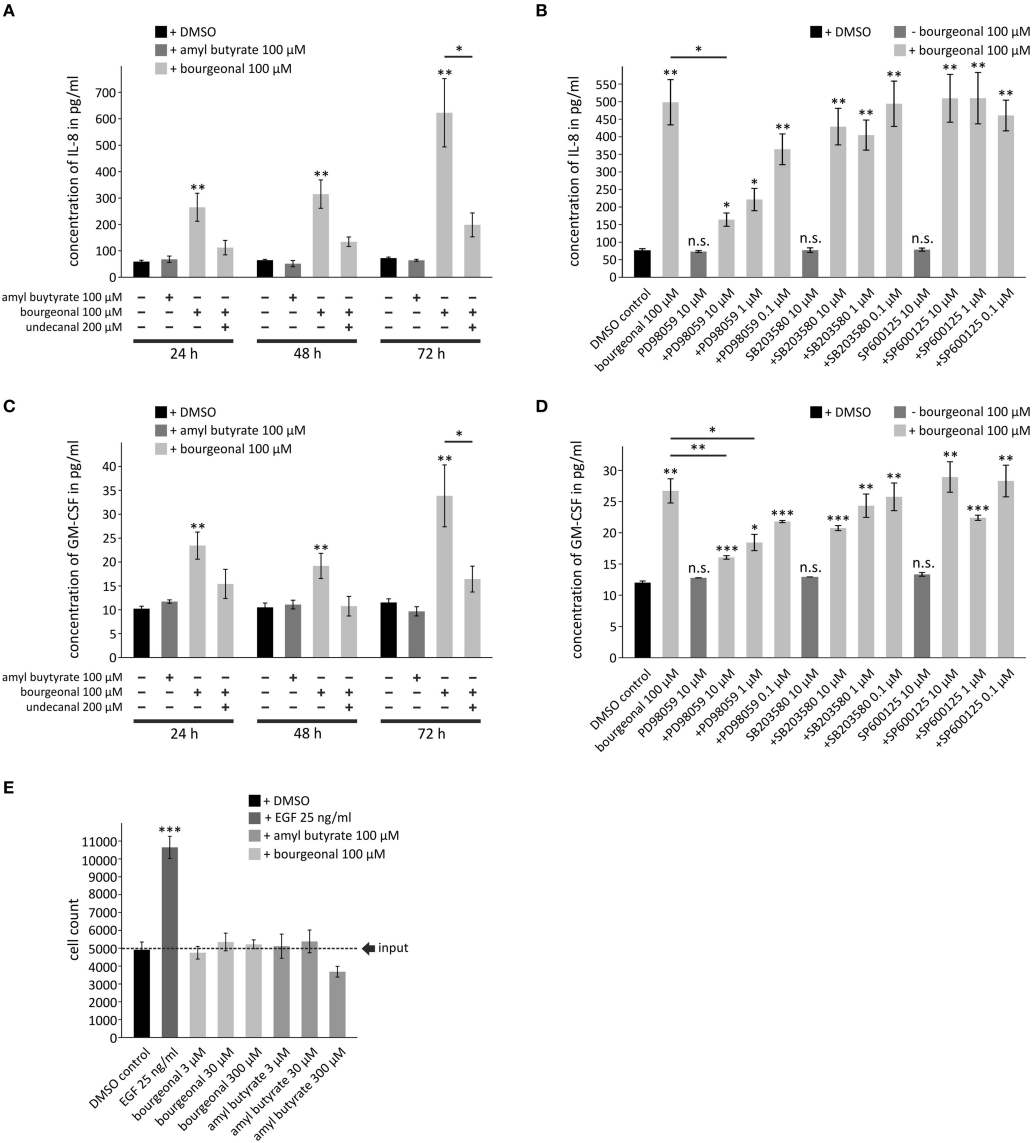
**Bourgeonal increases the production of inflammatory cytokines in HASMCs**. Serum-deprived HASMCs were stimulated with bourgeonal or amyl butyrate in the presence or absence of undecanal, the ERK inhibitor PD98059, the p38MAPK inhibitor SB203580, or the Jnk inhibitor SP600125 at the indicated concentrations and for the indicated times **(A,C)**, for 72 h **(B,D)** or for 6 days **(E)**. **(A–D)** The cytokines in the cell culture supernatants were measured by ELISA. **(E)** Viable cells were counted by Trypan blue staining. Stimulation with EGF was used as a positive control. Data **(A–D**, *N* = 4; e, *N* = 3) are presented as mean ±SEM. Significance was tested with an unpaired two-sample Student's *t*-test. ^*^p < 0.05, ^**^p < 0.01, and ^***^p < 0.001 vs. non-stimulated controls if placed on top of the bars or to values as indicated. n.s., differences to non-stimulated controls are not significant.

Finally, we investigated whether OR activation might lead to cellular responses that are directly associated with airway inflammation and remodeling. We analyzed the production of cytokines that play key roles in chronic inflammatory airway diseases, cell counts (indicates proliferation), and cell death. Bourgeonal but not amyl butyrate induced the production of IL-8 and GM-CSF in HASMCs in a time-dependent manner. Maximum secretion was found after 72 h of stimulation (Figures 8A,C). The effects of bourgeonal were clearly reduced by undecanal and by the ERK MAPK inhibitor PD98059 but not by inhibitors of p38 MAPK (SB203580) or Jnk (SP600125; Figures 8B,D). This behavior indicates that OR1D2 transmits signals via the ERK pathway to regulate IL-8 and GM-CSF production in HASMCs in response to bourgeonal.

Neither bourgeonal nor amyl butyrate significantly affected the cell counts after 6 days of incubation, although the highest concentration of amyl butyrate tended to reduce cell counts (Figure 8E). This finding indicates that neither odorant dramatically influences cell proliferation or death. The data further show that the upregulation of cytokine release by odorants did not result from an increase in the number of cells but from an increase in the production of cytokines in single cells.

## Discussion

In this study, we demonstrated that the human ORs OR2AG1 and OR1D2 are functionally expressed in HASMCs at the RNA and protein levels and are ubiquitously present in the bronchial tissue. The expression of ORs as specialized chemosensors in tissues and cells outside the nose has been previously reported (Feldmesser et al., 2006; Flegel et al., 2013). However, only a few studies have investigated the physiological impact of these ectopically expressed ORs. We showed for the first time that the activation of OR2AG1 by its specific ligand amyl butyrate (Neuhaus et al., 2009) and of OR1D2 by different known ligands (Spehr et al., 2003) both induced a transient Ca^2+^ increase in HASMCs. The specific activation of OR1D2 was verified by the application of the known antagonist undecanal (Spehr et al., 2003), which significantly reduced the bourgenol-elicited Ca^2+^ signals in HASMCs. Specific antagonists have previously been demonstrated to be reliable tools for validating OR activation (Spehr et al., 2003; Oka et al., 2004; Neuhaus et al., 2009). In addition, we found that both receptors trigger a cAMP-dependent signal transduction cascade, including the activation of adenylyl cyclase, generation of Camp, and opening of the CNG channel. Interestingly, we detected the olfactory signaling proteins ACIII, G_olf_, CNGA2, and CNGA4 in western blot and immunohistochemical experiments, further indicating that olfactory signaling in HASMCs depends on cAMP. The involvement of olfactory proteins in cellular signaling within non-chemosensory tissues has been demonstrated in keratinocytes (Busse et al., 2014) and the hepatocellular carcinoma cell line Huh7 (Maßberg et al., 2015), and these signals initialized diverse physiological reactions. Generally, HASMCs require increased intracellular Ca^2+^ levels to trigger a contraction. However, most studies of HASMCs have focused on Ca^2+^ release from intracellular stores, like the sarcoplasmic reticulum, and not on extracellular Ca^2+^ influx, as observed in cardiac, vascular, and skeletal muscle cells. Nevertheless, we here present a novel molecular mechanism that leads to elevated cytosolic Ca^2+^ concentrations via chemosensory GPCR, adenylyl cyclase, and cAMP generation as well as CNG channels. We observed an inhibitory effect of bourgeonal and amyl butyrate on the histamine-induced Ca^2+^ increase and speculate a potential crosstalk between the underlying signaling pathways. Histamine is known to activate a phospholipase C-mediated pathway and a subsequent release of Ca^2+^ from intracellular stores (Kotlikoff et al., 1987; Murray and Kotlikoff, 1991). Another product of the phospholipase C, diacylglycerol, can inhibit CNG channel opening (Crary et al., 2000). This could be one possible inference between histamine- and bourgeonal-induced intracellular Ca^2+^ responses. The importance of cAMP as a secondary messenger molecule in HASMCs and a therapeutic target for asthma and COPD patients has already been demonstrated with regard to β_2_-adrenoreceptors (Billington et al., 2012). In this context, cAMP antagonizes HASMC contraction, inhibits cell proliferation and migration, and combats the pathophysiology of airway narrowing and remodeling. Furthermore, solitary pulmonary neuroendocrine cells reportedly express different ORs and can respond to inhaled volatile chemicals via the secretion of distinct neuropeptides. Because COPD patients exhibited altered chemoresponsiveness, these findings may help to clarify odorant-induced airway reactions (Gu et al., 2014).

Remarkably, we found that OR1D2 and OR2AG1 specifically trigger odor-enhanced or odor-inhibited cell contraction, respectively, although both act on a cAMP-dependent signaling pathway. Generally, HASMC contraction is mediated by an increase in the cytosolic Ca^2+^ concentration and leads to the phosphorylation of myosin, whereas dilatation depends on cAMP and inhibits myosin phosphorylation (Gao et al., 2013). The activation of OR2AG1 leads to a decrease of the histamine-induced contraction via an AC-dependent signaling pathway. Thus, our data indicate that OR2AG1 activation via the volatile ligand amyl butyrate might constitute a novel therapeutic target to achieve smooth muscle dilatation in chronic inflammatory airway disease patients. Further, we speculate that the particular ORs might initiate different down-stream signaling molecules, such as protein kinase A [relaxation (Murthy et al., 2003)], Epac [relaxation (Roscioni et al., 2011)], protein kinase C [contraction (Walsh et al., 1994)], or guanine nucleotide exchanging factors [RhoGEFs; contraction (Artamonov et al., 2013)], thereby regulating further cellular responses. The bourgeonal-mediated contraction of HASMCs seems to be dependent on cAMP signaling, but does not depend on the opening of CNG channels. Beside the aforementioned pathways, Ca^2+^-independent contraction can be modulated and initiated by differences in the MCLK gene expression (Benayoun et al., 2003) or Ca^2+^-independent Rho kinase signaling (Schaafsma et al., 2008; Chiba et al., 2010). Moreover, complex spatio-temporal compartmentalized cAMP signaling networks have been shown to correlate with altered physiological output and the onset of chronic obstructive inflammatory diseases (Dekkers et al., 2013). Interestingly, the histamine-induced contraction is interfered by the co-stimulation of bourgeonal. The complexity of OR-signaling was demonstrated in a previous study, where it has been shown that a murine OR can simultaneously bind two different G proteins (G_olf_ and G_o_). In this context, two different odorants trigger completely independent pathways after activating the same OR *ex vivo* (Scholz et al., 2016). This dual capacity is discussed for GPCRs in general (Gurwitz et al., 1994; González-Maeso and Sealfon, 2012; Zocher et al., 2012). In addition, the extracellular environment has been shown to influence contractile gene expression in SMCs and thus phenotypic changes, such as contraction. For example, plating SMCs on collagen IV resulted in elevated contractile gene expression, whereas polymerized collagen I, which was used in our study, inhibited the expression of these genes (Koyama et al., 1996; Orr et al., 2009). *In vitro* gel contraction analysis revealed that the phenotype of cells isolated from patients with asthma is preserved and show a hypercontractility to the constrictor histamine (Matsumoto et al., 2007). Lung diseases like asthma affect the extracellular matrix remodeling of HASMCs. It was shown that this effect on the remodeling can also be analyzed by collagen gel experiments in long-term stimulus (72 h) experiments (Bourke et al., 2011). The slowly decreasing gel diameter is not associated with contraction. However, we observed a fast (<30 min) and strong effect on the gel diameter upon histamine and bourgeonal application, which highlights their contraction-inducing properties. In general, a detailed molecular analysis of potential down-stream key elements and the implications to asthma/COPD should be conducted in future studies with cells or tissues of diseased patients.

Evidence suggests that perfumes can exacerbate asthma (Kumar et al., 1995; Berger et al., 2006; Henneberger, 2007). Exacerbations are an acute worsening of symptoms that often lead to hospitalization and trigger disease progression. Pathophysiologically, asthma is characterized by chronic airway inflammation that increases in severe and exacerbated asthma, and these changes are associated with IL-8 and GM-CSF hyperproduction in immunoactive airway cells (Barnes, 2008). We have recently shown that HASMCs contribute to chronic airway inflammation in asthma, rendering them an auspicious cellular therapeutic target (Knobloch et al., 2010, 2013). Here, we demonstrated that bourgeonal (synthetic odor for lily-of-the-valley, commonly used in perfumes) induces IL-8 and GM-CSF release from HASMCs. These data are the first to indicate the influence of an odorant on the production of inflammatory cytokines in a lung cell type. Because bourgeonal did not significantly influence HASMC proliferation, we can exclude that effect of changes in cell number on the cytokine concentration; instead, these changes are only due to changes in secretion. Our data showing that undecanal and PD98059 reduce cytokine production in bourgeonal-exposed HASMCs provide a first indication that OR1D2 antagonists and ERK-inhibitors might be useful in the context of chronic inflammatory airway diseases. IL-8 and GM-CSF also contribute to airway inflammation in stable and exacerbated COPD (Barnes, 2008). Thus, our data suggest that exposure to distinct odorants might influence COPD and asthma pathogenesis.

Taken together, we here describe new molecular chemosensory mechanisms that affect important (patho-)physiological properties in HASMCs. Importantly, we modulated cell contraction and the secretion of cytokines by specifically activating OR2AG1 and OR1D2, respectively. We observed that the OR1D2 agonist bourgeonal has acute effects (fast transient Ca^2+^ increase and contraction) and a chronic effect (secretion of cytokines). The underlying pathways of OR2AG1 and OR1D2 activation and the modulation of the contractility of HASMCs needs to be further elucidated. We speculate that ORs might have implications on chronic inflammatory airway diseases and conclude that studies with human subjects could strengthen our hypothesis in future.

## Author contributions

Conception and design: BK, JK, AK, HH, and SO; Analysis and interpretation: BK, JK, VS, CW, SP, MS, ES, PS, FJ, and SO; Drafting the manuscript for important intellectual content: BK, JK, EH, HL, AK, HH, and SO

### Conflict of interest statement

The authors declare that the research was conducted in the absence of any commercial or financial relationships that could be construed as a potential conflict of interest.
