# Supplementary material for: Olfactory Receptors Modulate Physiological Processes in Human Airway Smooth Muscle Cells
Source: Front Physiol. 2016 Aug 4;7:339. doi: 10.3389/fphys.2016.00339 (PMC4972829; doi:10.3389/fphys.2016.00339)
Supplement: Supplementary file 1 [file DataSheet1.DOCX]

**Supplementary Figure legends**

**
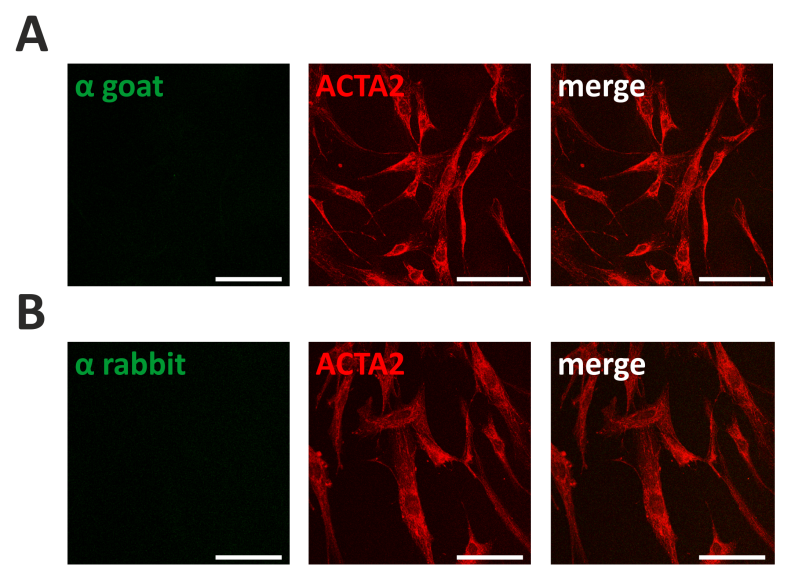
**

**Suppementary Figure 1** Goat anti-rabbit (A) and rabbit anti-goat (B) secondary antibody controls of fixed HASMCs. After the blocking step, cells were incubated another 4 h with blocking solution and washed in PBS buffer. Images were captured on the same conditions as Figure 2. Secondary antibody (α-rabbit 488 nm, green) was incubated on fixed cells for 45 min. Cells were then washed and embedded in mounting medium. DAPI was used to visualize cell nuclei (blue). No unspecific fluorescence could be observed in the 488 nm channel. Scale bars: 100 µm.

**
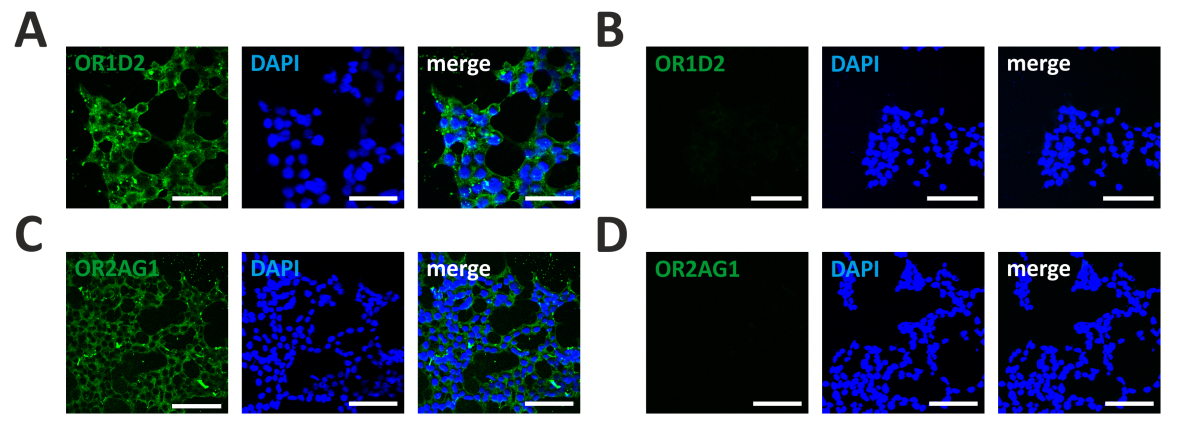
**

**Supplementary Figure 2** OR1D2 and OR2AG1 antibody specificity tests of transfected HANA3A cells. (A, C) HANA3A cells were transfected with either an OR1D2- (A) or OR2AG1- (B) carrying plasmid. Fixed cells were incubated with either OR1D2 or OR2AG1 antibody and subsequently immunhistochemical detection was conducted as described before. OR-staining was observed in transfected cells (green). To visualize the cell nucleus, DAPI was used. (B, D) Immunhistochemical staining of mock-transfected HANA3A cells with OR1D2 (B) and OR2AG1 (D) antibody. Scale bars: 50 µm (A), 100 µm (B, C, D).


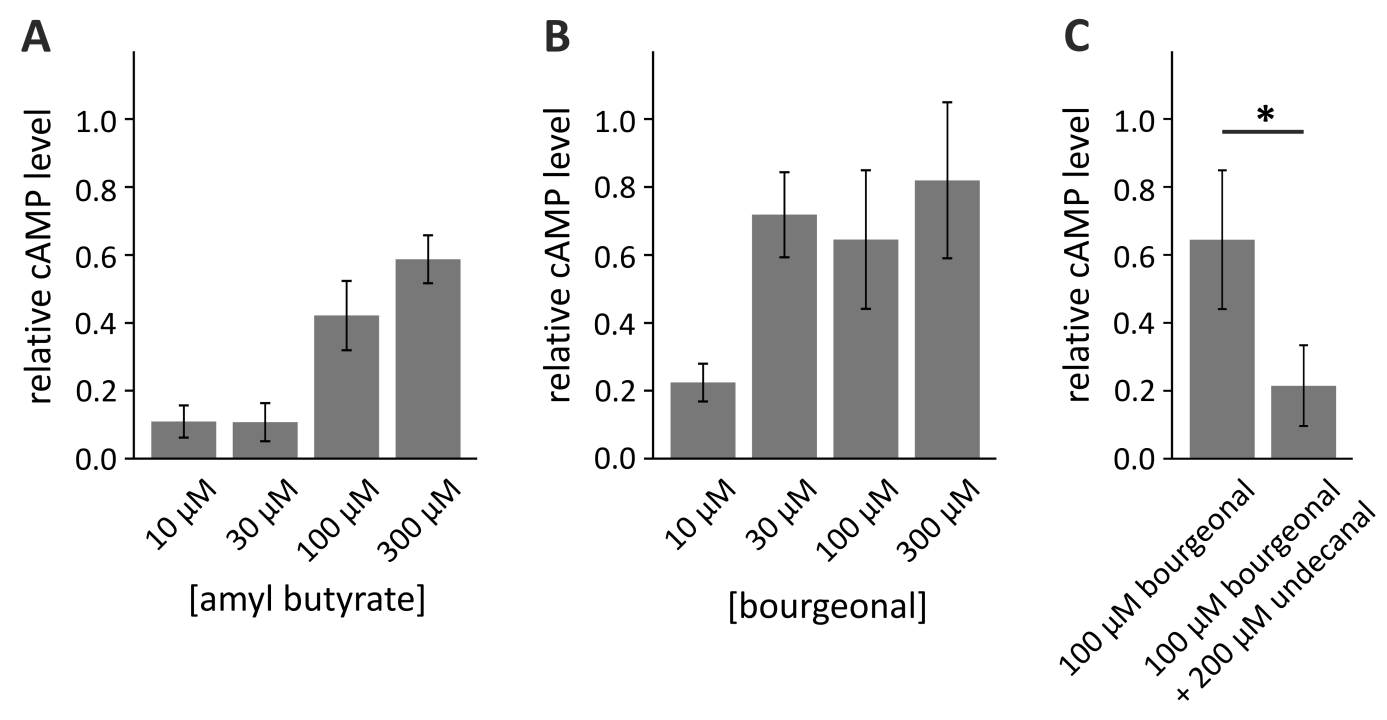


**Supplementary Figure 3** cAMP assay of HASMCs stimulated with odorants. The cAMP levels were normalized to 0.1% DMSO. (A, B) Stimulation with amyl butyrate (A) (10 µM, 30 µM, 100 µM, and 300 µM) (N = 4) and bourgeonal (B) (10 µM, 30 µM, 100 µM, and 300 µM) (N = 4) led to an increase in the relative cAMP level. (C) Co-incubation of bourgeonal (100 µM) with undecanal (200 µM) led to a significant inhibition of bourgeonal-induced elevation of the cAMP level (N = 4). Data are presented as mean ±SEM. Significance was tested with an unpaired two-sample Student’s t test. *p<0.05


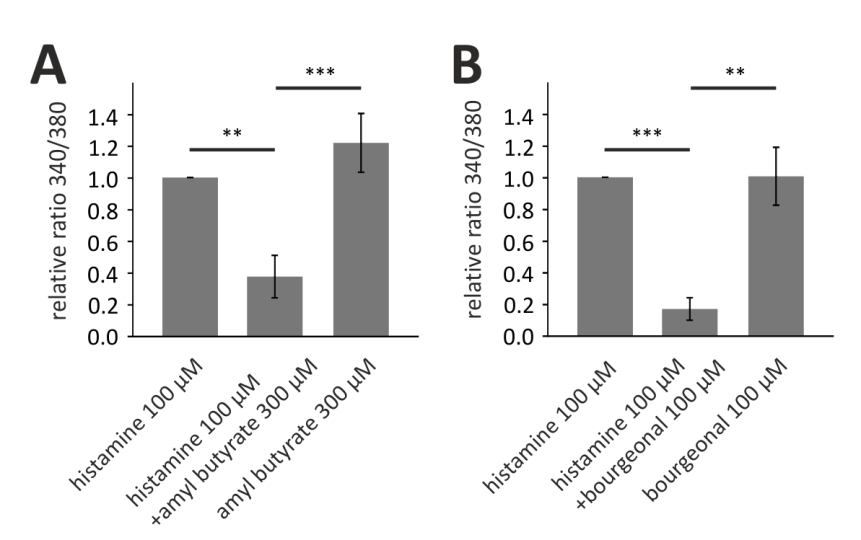


**Supplementary Figure 4**

Ca^2+^ imaging experiments of HASMCs stimulated with amyl butyrate (300 µM), bourgeonal (100 µM), and histamine (100 µM). Histamine was applied for 30 s on HASMCs (A, B). After 1 min wash out with extracellular solution, histamine was co-applied with either amyl butyrate (A) (N = 11) or bourgeonal (B) (N = 7). At the end of the measurement, amyl butyrate (A) or bourgeonal (B) were applied alone. The ratio of the amplitudes were normalized to the histamine-induced amplitude. Data are presented as mean ±SEM. Significance was tested with an unpaired two-sample Student’s t test. **p<0.01, ***p<0.001
